# Analysis of the spatio-temporal network of air pollution in the Yangtze River Delta urban agglomeration, China

**DOI:** 10.1371/journal.pone.0262444

**Published:** 2022-01-11

**Authors:** Chuanming Yang, Qingqing Zhuo, Junyu Chen, Zhou Fang, Yisong Xu

**Affiliations:** 1 School of Business, Suzhou University of Science and Technology, Suzhou, Jiangsu Province, China; 2 College of Management and Economics, Tianjin University, Tianjin, China; 3 Business School, Hohai University, Nanjing, Jiangsu Province, China; China University of Mining and Technology, CHINA

## Abstract

The complex correlation between regions caused by the externality of air pollution increases the difficulty of its governance. Therefore, analysis of the spatio-temporal network of air pollution (STN-AP) holds great significance for the cross-regional coordinated governance of air pollution. Although the spatio-temporal distribution of air pollution has been analyzed, the structural characteristics of the STN-AP still need to be clarified. The STN-AP in the Yangtze River Delta urban agglomeration (YRDUA) is constructed based on the improved gravity model and is visualized by *UCINET* with data from 2012 to 2019. Then, its overall-individual-clustering characteristics are analyzed through social network analysis (SNA) method. The results show that the STN-AP in the YRDUA was overall stable, and the correlation level gradually improved. The centrality of every individual city is different in the STN-AP, which reveals the different state of their interactive mechanism. The STN-AP could be subdivided into the receptive block, overflow block, bidirectional block and intermediary block. Shanghai, Suzhou, Hangzhou and Wuxi could be key cities with an all above degree centrality, betweenness centrality and closeness centrality and located in the overflow block of the STN-AP. This showed that these cities had a greater impact on the STN-AP and caused a more pronounced air pollution spillovers. The influencing factors of the spatial correlation of air pollution are further determined through the quadratic assignment procedure (QAP) method. Among all factors, geographical proximity has the strongest impact and deserves to be paid attention in order to prevent the cross-regional overflow of air pollution. Furthermore, several suggestions are proposed to promote coordinated governance of air pollution in the YRDUA.

## 1. Introduction

With the rapid development of industrialization and urbanization, China’s economy has achieved rapid growth, but the country has suffered serious air pollution [1, 2]. To realize the coordination between economic growth and ecological governance, in *the Fourteenth Five-Year Plan*, China clearly defined air pollution prevention and control actions that must be carried out to eliminate heavy pollution weather. Air pollution is a typical public problem with characteristics such as cross-regional, strong fluidity, wide influence, noncompetitive and nonexclusive, and it usually formed a correlation within a certain region [3]. These make the effect of the fragmented governance that depends on administrative divisions very limited. It is objectively required to improve regional coordinated governance of air pollution.

As one of the most developed regions in China, the Yangtze River Delta urban agglomeration (YRDUA) has suffered significant air issues [4, 5], and it has been listed as a key area for air pollution control in China. Facing resource utilization, city construction and economic development, cities are unwilling to take responsibility for the negative externalities of air pollution, mostly because they selfishly pursue their own economic growth and environmental statues. To improve coordinated governance of the YRD, in November 2018, China officially identified the integrated development of the YRD as a national strategy. And in December 2019, the *Outline of the Integrated Regional Development of the Yangtze River Delta* was promulgated. This policy alleviate the conflict of motivations for air pollution control and enhance the awareness of cooperation between cities, which provides an excellent opportunity for the coordinated governance of air pollution in the YRD.

Recently, the spatio-temporal distribution and regional correlation of air pollution have received extensive attention worldwide. Some studies have simulated the cross-regional dynamic diffusion and transmission process of air pollution based on physical or chemical methods. Researches concluding that the air pollution in a region will be imported from its environs, then be transported to others, and ultimately forming a pollution cycle and causing more serious pollution [6, 7]. Several studies prove that mobile monitoring across multiple census tracts can quantitatively visualize the spatial distribution patterns of air pollution [8, 9]. Currently, some scholars finding that the spatial correlation of air pollution between regions have formed a spatial network structure [10–13]. However, the study that analyze the structural characteristics formed by the spatio-temporal correlation of air pollution is relatively rare.

Studies on the influencing factors of air pollution can be classified into two main aspects: socio-economic factors and natural ones. Firstly, air pollution is highly correlated with economic development [14]. Industrial pollution sources are positively correlated with air pollution [15]. In terms of natural conditions, there is a strong intercorrelation between air pollution and temperature [16]. Unusual precipitation increases the amount of chloride ions in the air, showing a negative impact on air pollution [17]. The impact of socio-economic factors is more significant than that of natural ones [18]. However, relying only on the research on the influencing factors of the air pollution level is not enough to support the decision-making of air pollution control at the regional scale. It is urgent to identify the spatial correlation characteristics of air pollution between regions to realize the coordinated governance of regional air pollution.

Therefore, in this study, (1) The spatial correlation of air pollution is identified by an improved gravity model, and the STN-AP in the YRDUA is visualized by *UCINET*. (2) The characteristics of the STN-AP are revealed by social network analysis (SNA) method. (3) The key cities of the STN-AP are furtherly determined by the classification of individual and clustering characteristics. (4) Based on quadratic assignment procedure (QAP) method, the socio-economic and natural factors influencing the spatial correlation of air pollution are analyzed. At last, several suggestions are proposed to strengthen the coordinated governance of air pollution in the YRDUA.

## 2. Methods and data

### 2.1 Study area

The YRDUA includes 27 core cities as shown in S1 Table. In 2020, the population and GDP of the YRD accounted for 16.66% and 24.09% of China, respectively. The annual average number of days of air pollution (days that the air quality index, AQI, exceed 100) in the YRD was 55, accounting for 15.07% of the whole year. Meanwhile, the annual average concentrations of PM_2.5_, NO_2_, O_3_ in this region was 35mg/m^3^, 29 mg/m^3^ and 152 mg/m^3^ respectively, exceeding the average level of China by 2 mg/m^3^, 5 mg/m^3^ and 14 mg/m^3^ respectively. The high frequency of air pollution and the strong concentration of pollutants make the YRD the region with one of the most serious air pollution in China and seriously violating the goals of ecological sustainable development.

### 2.2 Identifying the spatial correlation of air pollution

Many modeling methods can be used to determine spatial correlation, such as vector autoregression model and gravity model [19–21]. However, the vector autoregression model is too sensitive to the lag order, which will reduce the accuracy of the network correlation. The gravity model is not only suitable for total data but also can comprehensively consider factors such as economic development and geographical distance. Therefore, this study chooses the gravity model to construct the STN. The traditional gravity model is as follows.


$$R_{ij}=k_{ij}\frac{L_{i}L_{j}}{D_{ij}{}^{2}}$$ (1)

*R*_*ij*_ represent the gravity between cities *i* and *j*. *L*_*i*_ and *L*_*j*_ are attribute index of cities *i* and *j* respectively. *k*_*ij*_ and *D*_*ij*_ are the gravitational coefficient and the distance between the cities respectively.

Energy consumption is the main source of air pollution because a large amount of carbon dioxide, sulfur dioxide, soot, etc., which would form haze and acid rain. GDP and population reflect the economic development level and size of a city respectively, and they are strongly related to air pollution [22]. Single pollutant or the air pollution index (API) are often used as an indicator to study air pollution and its mutual effects occurring in regions [23–25]. With the development of air po1llution monitoring technology, three pollutants, i.e., PM_2.5_, O_3_, and CO, have been added to the API to form the AQI. The AQI as an air pollution indicator can more fully reflect the air pollution situation and make the research results more reliable. At the same time, the research indicator are processed by geometric average in the gravity model [26, 27], which can quantify the comprehensive effect of indicators, also can consider the cross-effects of each indicators between regions. Therefore, we use the geometric average method to incorporate the above-mentioned air pollution indicators into the gravity model. The ratio of the AQI of a city to the total AQI to show the gravitational coefficient. The improved gravity model is as follows.


$$R_{ij}=k_{ij}\frac{\sqrt[{4}]{P_{i}G_{i}Q_{i}E_{i}}\times \sqrt[{4}]{P_{j}G_{j}Q_{j}E_{j}}}{D_{ij}{}^{2}},\;k_{ij}=\frac{Q_{i}}{Q_{i}+Q_{j}}\;(i=1,2,\ldots ,n;j=1,2,\ldots ,m)$$ (2)

*R*_*ij*_ is the spatial correlation of air pollution between city *i* and *j*. *Q*_*i*_, *P*_*i*_ (unit: 10,000 people), *G*_*i*_ (unit: 100 million yuan) and *E*_*i*_ (unit: 10,000 tons of standard coal) represent the AQI of city *i*, the total population at the end of the year, GDP and energy consumption, respectively. Furthermore, energy consumption is expressed by the total energy consumption of industrial enterprises above a designated size. *k*_*ij*_ and *D*_*ij*_ (unit: km) are air pollution adjustment coefficient and spherical distance between cities, respectively.

Gravity model also has defects, that is, the strength of a correlation cannot be directly derived. The average value of a correlation is typically used as the critical value to judge the strength of the correlation [12, 28], but this method is relatively subjective, which may lead to errors in the results. To compensate for this defect, we adopted a relatively objective method, the Weaver index [29]. In a multifactor system, the Weaver index is an effective tool for determining significant relationships. It establishes an approximate hypothesis to test by comparing the actual distribution with the hypothetical distribution. The main steps are as follows.

Step 1: Set *n* samples and *m* types of indicators, where *a*(1,*j*),…,*a*(*n,j*) are *n* sample values corresponding to the *j* index. After sorting the sequence from large to small, we can obtain *A*(1,*j*),*A*(2,*j*),…*A*(*n,j*).

Step 2: Calculate the Weaver index *w*(*i,j*) of sample *i* under index *j*.


$$w(i,j)={\sum_{i=1}^{n}\left[s(k,i)-100A(i,j)/\sum_{i=1}^{n}A(i,j)\right]}^{2}$$ (3)

$$s(k,i)=\{\begin{array}{l} 100/i,(k\leq i)\\ 0,(k>i) \end{array},\;(i=1,2,\ldots ,n;j=1,2,\ldots m)$$ (4)

Step 3: Set the main samples as *u* = {*u*|*w*(*u,j*) = min*w*(*i,j*), (*i* = 1,2,…,*n*)}, and the main sample set as *X_j_* = {*p|p* = 1,2,…,*u*}. The index value *A*(*u,j*) of the *u* sample is the critical value determined by the Weaver index. The *X*_*j*_ sample above the critical value is an important sample.

### 2.3 Selecting the network characteristics index

SNA is a quantitative analysis method that combines graph theory with mathematical methods, it can effectively measure and analysis the network characteristics [30]. SNA has been used to describe the characteristics of the air pollution network [31, 32]. In our paper, the 27 core cities in the YRDUA are used as the network nodes, and the spatial correlation of air pollution identified above is used as paths to build social network. Furtherly, the network characteristics are analyzed by the network characteristic index. The analysis index of network characteristics mainly includes the overall network characteristics, individual network characteristics, and network clustering characteristics [33]. Among them, the overall network characteristics are described by network density (*M*_*1*_), network relevance (*M*_*2*_), the network hierarchy (*M*_*3*_), and network efficiency (*M*_*4*_). *M*_*1*_ indicates the closeness of the correlation among cities in the STN. *M*_*2*_ reflects the reachability and robustness of the STN. *M*_*3*_ and *M*_*4*_ represent the hierarchical structure of each city and the degree of redundant correlation in the STN, respectively. The individual network characteristics are qualified by degree centrality (*N*_*1*_), betweenness centrality (*N*_*2*_) and closeness centrality (*N*_*3*_). *N*_*1*_ denotes the extent to which cities are centrally located in the STN. *N*_*2*_ shows the degree to which a city controls other cities. *N*_*3*_ indicates the ability of a city to directly connect with other cities. The network clustering characteristic use the convergent correlations [34] method to divide the STN into several different blocks based on certain rules, and then to analyze the role of each block and the relationships between the blocks in the STN.

### 2.4 Data sources

This study constructs STN-AP based on air pollution data of 27 core cities in the YRDUA from 2012 to 2019. The data sources are shown in Table 1.

**Table 1:** Data sources.

Data	Unit	Type	Source
AQI	\	txt	http://www.mee.gov.cn
Population	10,000 people	txt	The statistical yearbook and development bulletin of each city
Energy consumption	10,000 tons of standard coal	txt	
GDP	100 million yuan	txt	
Urbanization level	%	txt	
Industrial structure	%	txt	
Car ownership	10,000 vehicles	txt	
Precipitation	Millimeter	txt	http://data.cma.cn/data/weatherBk.html
Temperature	Celsius	txt	
Sunshine hours	Hour	txt	
Spherical distance	Kilometer	txt	Google Maps software

## 3. Results and discussion

### 3.1 Spatio-temporal distribution of air pollution in the YRDUA

**Fig 1:**
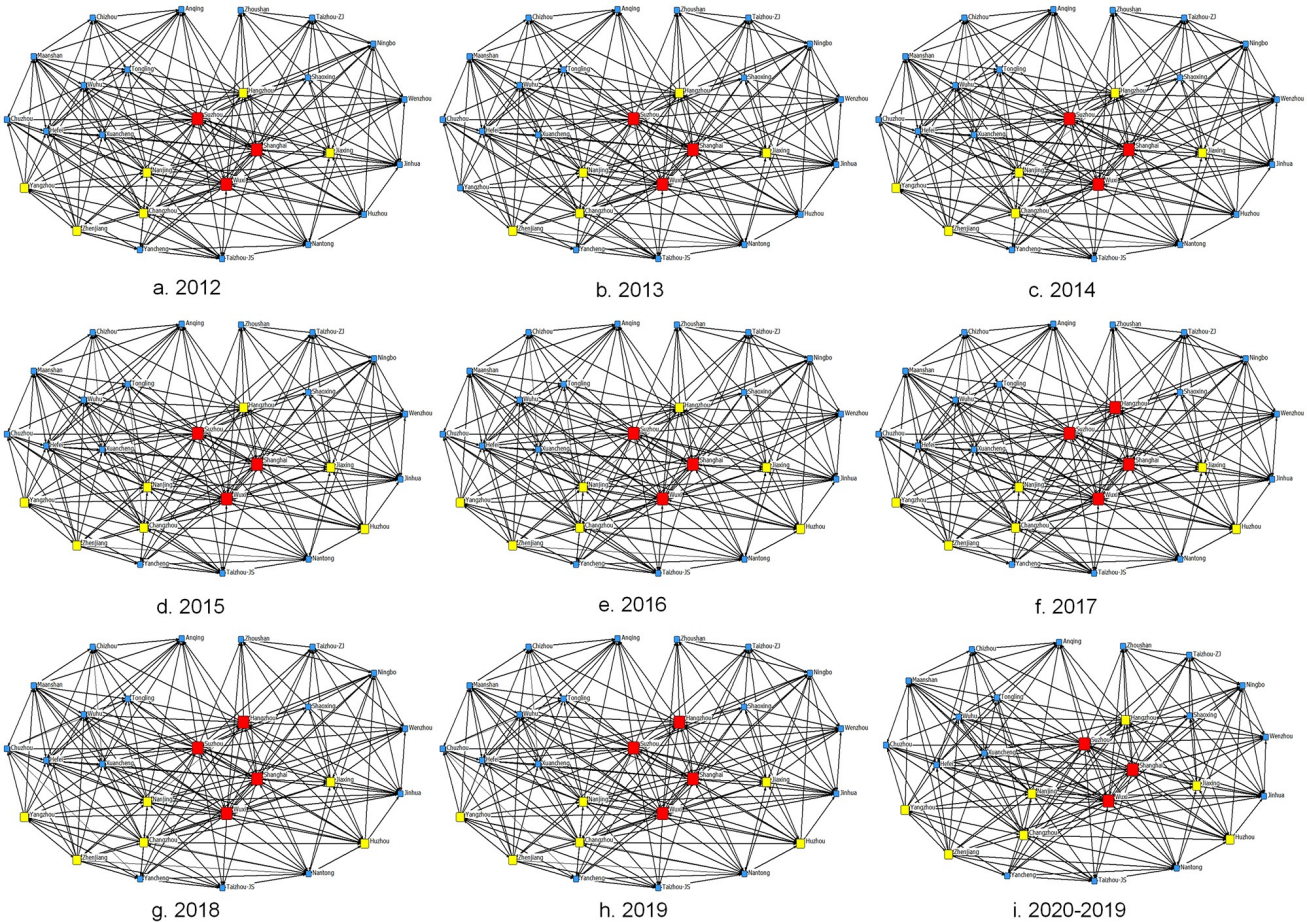
Spatio-temporal network of air pollution in the YRDUA. Since the number of outgoing relationships in each city ranges from 0 to 30, cities with more than 20 outgoing relationships are marked in red, those with between 10 and 20 are marked in yellow, and the others are marked in blue.

This study identifies the spatial correlation of air pollution in the YRDUA through an improved gravity model, and the STN-AP from 2012 to 2019 is visualized using *UCINET 6*.*0* (see Fig 1).

Overall, the spatial correlation of air pollution in the YRDUA displays a typical "central–marginal" network structure. Hangzhou, Shanghai, Wuxi, and Suzhou located at the center of the STN-AP. There is no isolated city in the network, indicating that air pollution of a city is not only related to its economic development and population, but also affected by air pollution in other cities. Additionally, connections of cities changed dynamically during the study period. Specifically, after 2016, Hangzhou changed from yellow to red, becoming the fourth city with more than 20 outgoing relationships. After 2014, Huzhou changed from blue to yellow, indicating that the number of outgoing relationships between Huzhou and other cities is increasing. Hangzhou and Huzhou are both located in the south of the YRDUA. While in 2013, Yangzhou dropped to blue, and in 2014, it rose to yellow. The reason is that Yangzhou lost contact with Xuancheng in 2013, which caused the number of outgoing relationships in Yangzhou to drop from 10 to 9, but in 2014, Yangzhou re-established contact with Xuancheng. In the above analysis, we only categorize the cities into three intervals based on the number of outgoing relationship between cities, but the role of each city in STN-AP needs to further study.

### 3.2 Characteristics of the STN-AP in the YRDUA

#### (1) Overall characteristics

The overall characteristics of the STN-AP in the YRDUA from 2012 to 2019 are calculated based on *UCINET 6*.*0* (see Table 2).

**Table 2:** Overall characteristics of the spatio-temporal network in the YRDUA.

Year	Actual relationship	*M*_*1*_	*M*_*2*_	*M*_*3*_	*M*_*4*_
2012	256	0.365	1	0	0.292
2013	257	0.366	1	0	0.289
2014	261	0.372	1	0	0.277
2015	264	0.376	1	0	0.268
2016	267	0.380	1	0	0.258
2017	265	0.378	1	0	0.265
2018	266	0.379	1	0	0.262
2019	263	0.375	1	0	0.260
Average	262	0.374	1	0	0.271

Calculated by 27×(27–1), the maximum number of theoretical relationships of the whole network is 702, but the actual annual average number of relationships from 2012 to 2019 is only 262 (see Table 2). It can be seen that many cities still have the possibility of connection in this STN-AP. Except for 2017 and 2019, the density of the STN-AP shows an upward trend. Both the yearly value and the annual average value of the network relevance of the STN-AP are 1, indicating that all 27 cities have air pollution connections. In terms of overall efficiency, more than 70% of the correlation in the STN-AP overlap, meaning that the STN-AP has good stability but its efficiency is not high. With the increase in the interactions of air pollution, there is no obvious network hierarchy, which means that the subordination between cities in the STN-AP is weak. Comprehensively, the STN-AP structure in the YRDUA has been gradually optimized, but there are still problems such as insufficient correlation and low network efficiency.

#### (2) Individual characteristics

Based on the individual characteristics of the STN-AP of the YRDUA (see S2 Table), the degree centrality of all 27 cities shows an obvious nonequilibrium situation. Nine cities have an above average level, accounting for 47.11%. This result indicates that they have more connections with other cities and are located at the center of STN-AP in the STN-AP. Among them, Suzhou, Wuxi, and Hangzhou are the top three cities and they are pivotal areas for other cities. However, Yancheng, Chizhou and Zhoushan are the bottom three cities, as they are less correlated with other cities and are located in marginal areas of the STN-AP. Six cities, accounting for 81.84%, have an above average betweenness centrality. Among them, Suzhou, Wuxi, and Hangzhou play a dominant role in the YRDUA as they exhibit strong control over the STN-AP. However, the betweenness centrality of Chuzhou, Chizhou and Zhoushan makes them the bottom three cities. Nine cities including Suzhou, Wuxi and Nanjing have an above average closeness centrality, illustrating that these cities have more direct correlation to other cities in the STN-AP. The reason is that most of these cities are developed and have a strong resource conversion ability, so they can quickly establish correlation with other cities in the STN-AP. Yancheng, Chizhou and Zhoushan have scarce closeness centrality. We found that for all three types of centralities, Suzhou, Wuxi, Hangzhou, Shanghai, Nanjing, and Changzhou are all above average in the STN-AP.

#### (3) Clustering characteristics

The 27 cities are divided into four blocks with a maximum segmentation depth of 2 (see S3 Table). In addition, the four blocks are defined as four types. The first type is the receptive block which receives more external relationships compared to the overflowing external relationships of the block. The second type is the overflow block which overflows more relationships to other blocks than it receives external relationships. The third type is the bidirectional block which exhibits both external relationships and internal reception relationships, and the internal members of this block have more relationships. The fourth type is the intermediary block which has more external relationships and internal relationships as well as closer relationships with the members belong to other blocks. After the blocks are divided, the degree of internal agglomeration and external overflow can be described by comparing the actual internal relationship ratio (*Y*) and the expected internal relationship ratio (*Z*) of the block. We set *λ* as the number of overflow relationships inside the block, *ε* as the total number of overflow relationships, while *θ* and *η* as the number of cities inside the block and the number of all cities, respectively. The calculation methods are as follows: *Y* = *λ*/*ε* and *Z* = (*θ*-1)/(*η*-1). The overflow relationships of each block are presented in S4 Table.

There are 264 relationships in the STN-AP. Among them, 126 relationships are within each block, and 138 relationships are distributed between the blocks. The actual internal relationship ratio (91%) of block I is greater than the expected internal relationship ratio (27%). The number of received relationships in the block is 93, but the total number of overflow relationships is only 44. Thus, the block is the receptive block, and it has strong pollution absorption capacity. The actual internal relationship ratio (37%) of block II is greater than the expected internal relationship ratio (19%). The number of relationships inside the block is 52, and the number of relationships outside the block is 69. Thus, the block is the intermediary block, and it plays the role of a connector in the STN-AP. The actual internal relationship ratio (33%) of block III is greater than the expected internal relationship ratio (23%). The block has a high number of overflow relationships as 122, but 63 received relationships. Thus, this block is the overflow block with a strong pollution spillover effect. Finally, the actual internal relationship ratio (71%) of block IV is greater than the expected internal relationship ratio (19%), and it has 28 overflow relationships and 57 received relationships. Thus, block IV is the bidirectional block, and its absorption pulling effect is greater than its spillover pushing effect.

### 3.3 Key cities of the STN-AP

**Fig 2:**
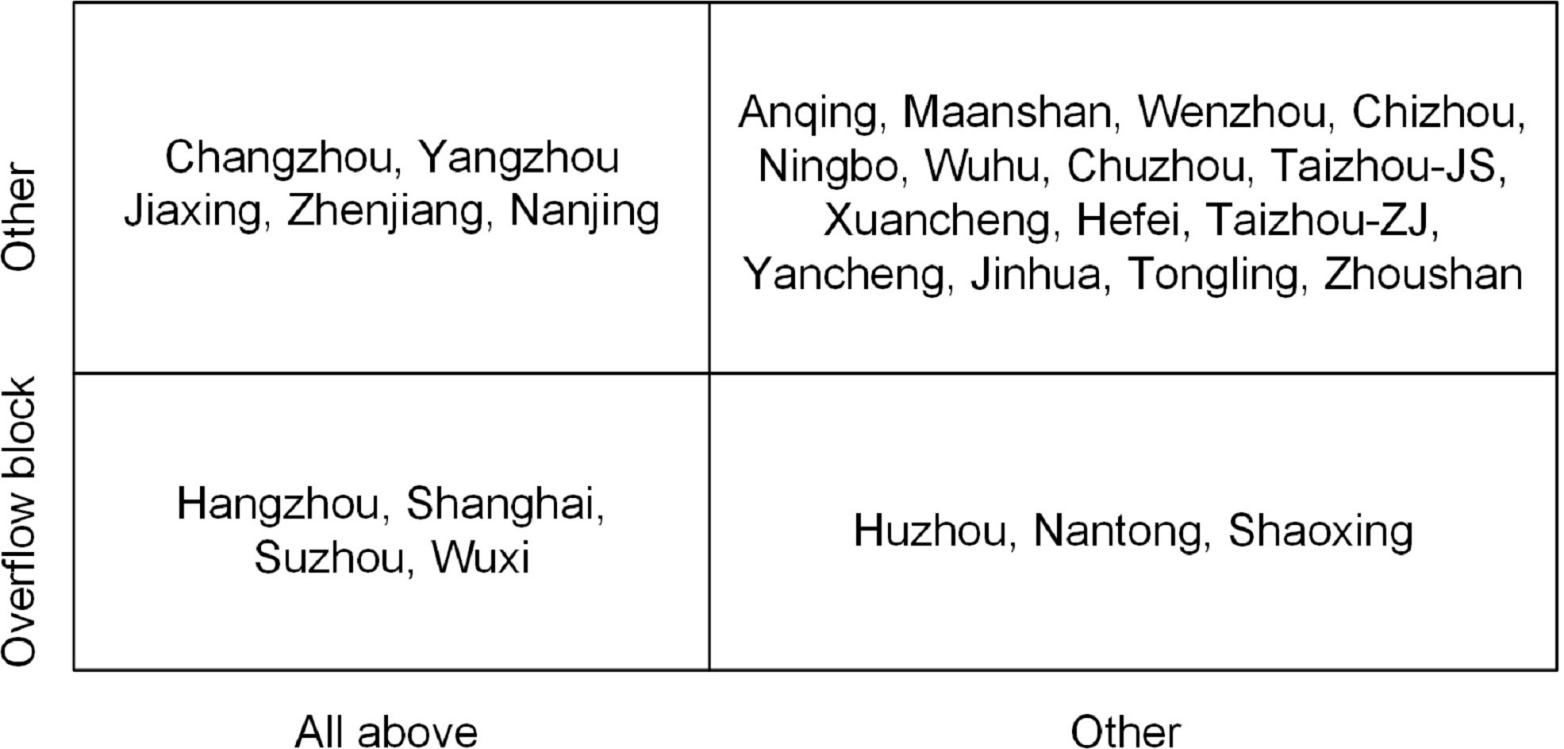
Classification of all above centrality and overflow block. All above mean that the city’s degree centrality, betweenness centrality and closeness centrality are all above the average level.

Regarding the individual characteristics of the STN-AP, we found that Suzhou, Wuxi, Hangzhou, Shanghai, Nanjing, and Changzhou are the cities with degree centrality, betweenness centrality and closeness centrality all above the average level. Compared with other cities, their changes in the population, economy, and energy consumption may cause more cities to change in the same way, however, they are not easily affected by other cities. At the same time, regarding the clustering characteristics of the STN-AP, the cities in the overflow block of the STN-AP not only have strong air pollution overflow capabilities but also jointly overflow with the cities in the block. This joint overflow is a difficult problem of air pollution control, because once a joint overflow is formed, these cities may have more pollution overflow to other cities than single city without joint. Considering the individual characteristics and clustering characteristics of the STN-AP, we classified the 27 cities into four quadrants based on whether they are cities with degree centrality, betweenness centrality and closeness centrality are all above the average level or not and whether they are located in the overflow block (Fig 2).

Wuxi, Suzhou, Shanghai, and Hangzhou are not only cities with degree centrality, betweenness centrality and closeness centrality are all above the average level in the STN-AP but also are located in overflow block. Thus, they could become the key cities of the STN-AP. Wuxi and Suzhou have serious air pollution, and Hangzhou and Shanghai have high population density [35], which may be a reason why they have become the key cities. On the one hand, key cities with great impact on the STN-AP can become the leaders, to be pioneers in formulating new air pollution policies and measures, then make improvements based on the implementation effects, and spread to others cities. At the same time, key cities can establish a joint mechanism for air pollution prevention and control to reduce their own pollution spillover and break the jointly overflow. On the other hand, based on inter-provincial administrative boundaries, key cities can be used as bridges to build inter-provincial connections to enhance the coordinated governance of the YRDUA. For each province, Shanghai can give full play to its advantages in air pollution control technological innovation to drive other cities. Jiangsu Province should continue to maintain the advantages of Wuxi and Suzhou in the environmental protection industry, and take them as the head to establish the environmental protection industry chain between cities, and further extend it to other regions. It also should help Anhui Province, which is geographically nearby, jointly with Shanghai and Zhejiang Province to establish a regional cooperation mechanism. Zhejiang Province may promote the formation of a pollution control circle in Hangzhou, Jiaxing and Huzhou and gradually expand the circle beyond the province.

### 3.4 Influencing factors of the spatial correlation of air pollution

Since the variables that influence the spatial correlation of air pollution are correlated data, the measurement methods traditionally to analysis the correlated data may have multi-collinearity problems, while QAP method does not need to consider the independence between variables, so it can overcome the problem [36, 37]. Therefore, this study uses the QAP method for correlation and regression analysis. We analysis the influencing factors of the spatial correlation of air pollution from following two aspects: (i) the socio-economic differences related to human activities determine the air pollution differences among cities, resulting in pollution overflow between cities and spatial changes in air pollution. Among all economic factors, the urbanization level has a strong impact on the spatial correlation of air pollution [38]. A change in the industrial structure reflects industrial optimization and technological upgrading, which is closely related to air pollution control. The increase in the number of motor vehicles will put tremendous pressure on urban traffic and air pollution, as manifested in congested roads and poor air flow. Additionally, the fine particles and nitrogen oxides in motor vehicle exhaust are important causes of haze. Therefore, we choose GDP (*G*), energy consumption (*E*), the urbanization level (*U*), the industrial structure (*I*) and car ownership (*C*) to measure socio-economic differences. Among them, the industrial structure is the ratio of the GDP of the tertiary industry to the regional GDP, and the urbanization level is the ratio of the urban population to the total population. (ii) Natural factors such as temperature, precipitation, and sunshine hours play an important role in the diffusion and accumulation of air pollution, resulting in changes in the spatial correlation [39]. Meanwhile, the air pollution of cities is also more susceptible to the impact of adjacent cities, which manifests as a spillover effect. Therefore, temperature (*T*), precipitation (*W*), sunshine hours (*S*), and geographical distance are used to measure the differences in natural factors. Among them, distances of [0, 200), [200, 400), and [400, 600) (unit: km) are selected to measure the proximity relationship, represented by *D*_*1*_, *D*_*2*_ and *D*_*3*_, respectively. The calculation results are shown in S5 and S6 Tables.

The correlation results show that all relevant indicators are significant, indicating that the regional differences in all of the factors have a strong influence on the spatial correlation of air pollution. Among them, the correlation coefficients of the differences in the GDP, energy consumption, urbanization level, industrial structure, temperature and distances less than 200 km of each city are positive, and the intensity order is *D*_*1*_> *E*> *C*> *G*> *I*> *U*> *T*. The correlation coefficients of the differences in precipitation, sunshine hours and distances above 200 km are negative, and the intensity order is *D*_*2*_> *D*_*3*_> *W*> *S*. Except for the differences in car ownership, sunshine hours, and distances above 200 km, the regression results for the other factors are significant, meaning that the differences in these factors have a strong influence on the spatial correlation of air pollution [13]. Among them, the regression coefficients of the differences in GDP, energy consumption, the urbanization level, the industrial structure, temperature and distances less than 200 km is positive, indicating that the difference in these factors between cities is greater, the intensity of their promotion is *D*_*1*_> *E*> *I*> *G*> *T*> *U*. Comparatively, the regression coefficients of the difference in precipitation is negative, indicating that the larger the difference in precipitation between cities, the smaller the intensity of the correlation. Among all the factors that influence the correlation of air pollution, distances less than 200 km, that is, geographical proximity, is the factor that has the greatest impact on the strength of correlation (0.513) and changes in differences (0.358). This is similar to the result that geographical distance can significantly affect the correlation of regional pollution control [28]. Therefore, cities should pay attention to their geographical proximity to prevent the overflow and spread of air pollution. For cities that have already caused air pollution overflow, appropriate economic or technical compensation should be provided to the cities that have received the overflow. Additionally, socio-economic factors such as energy consumption and the economic level also have a nonnegligible impact on the spatial correlation of air pollution. Compared with natural factors, such as temperature, the elasticity of socio-economic factors is relatively large, and the possibility that it will change is greater. Therefore, we suggest that the government should try to narrow the socio-economic differences between regions to accelerate the coordinated governance of air pollution. Specifically, the development of urbanization should be encouraged by the government. The regional energy consumption structure and industrial structure should be adjusted, and technology and capital in areas with a low economic level should be supported. In addition, urban energy consumption, car ownership and the urbanization level are all important aspects of environmental regulation, while environmental regulation is an important measure for controlling air pollution [40]. Therefore, we suggest that when formulating environmental governance policies, the government could improve laws and regulations concerned with regional air pollution control and regional ecological cooperation to realize the integrated high-quality development of the YRDUA.

### 3.5 Limitations

This study identified the spatial correlation of air pollution in YRDUA. However, only five socio-economic factors and four natural factors are analyzed. In view of the complex situation and the emerging factors, the corresponding indicators of spatial correlation of air pollution are far more than we consider. Additionally, the conflict of motivations for decision-making between cities are objectively existing in practice, but we have not yet estimate it in current study. Therefore, the conflict of motivations for decision-making and multi-factors affecting spatial correlation of air pollution would be analyzed in the future.

## 4. Conclusions

Based on the panel data of 27 core cities in the Yangtze River Delta urban agglomeration (YRDUA), we construct spatio-temporal network of air pollution (STN-AP) and reveal its characteristics, based on which, influencing factors of spatial correlation of air pollution are analyzed. Conclusions including: (1) the spatial correlation of air pollution in the YRDUA formed a complex STN-AP and displayed a typical "central–marginal" network structure. During the study period, the number of outgoing relationships of Hangzhou and Huzhou with other cities had gradually increased. (2) The STN-AP of YRDUA was relatively stable. Suzhou, Wuxi, Hangzhou, Shanghai, Nanjing, and Changzhou with degree centrality, betweenness centrality and closeness centrality all above the average level had a greater impact on the STN-AP. The STN-AP could be subdivided into the receptive block, overflow block, bidirectional block and intermediary block, and the cities in the overflow block caused air pollution spillovers to more cities. (3) After categorizing the individual characteristics and clustering characteristics of the STN-AP, we found that Wuxi, Suzhou, Shanghai, and Hangzhou could become the key cities with degree centrality, betweenness centrality and closeness centrality above average level and in the overflow block. The YRDUA should prevent air pollution in key cities from overflow to other cities, and take advantage of the greater impact of key cities in the STN-AP and regard key cities as leaders to guide other cities in planning air pollution control. (4) Socio-economic and natural factors have varying degrees of impact on STN-AP spillover in the YRDUA, and among these factors, geographical proximity has the greatest impact. Cities should prevent air pollution from geographical proximity cities from overflow, and can take economic or technical compensation measures for cities that have already overflowed. At the same, economic and social differences between regions should be narrowed and environmental regulations should be strengthened. In the context of the integration of the Yangtze River Delta, the urban agglomeration should pay more attention to the spatial relationships and joint control of air pollution.

## Supporting information

S1 Table 27 core cities in the YRDUA. (DOCX)

S2 Table Individual characteristics of the STN-AP in the YRDUA. (DOCX)

S3 Table The block classification. (DOCX)

S4 Table The spillover effect between blocks. (DOCX)

S5 Table QAP correlation results of influencing factors on spatial correlation of air pollution. (DOCX)

S6 Table QAP regression results of influencing factors on spatial correlation of air pollution. (DOCX)
