# Supplementary material for: Analysis of the spatio-temporal network of air pollution in the Yangtze River Delta urban agglomeration, China
Source: PLoS One. 2022 Jan 11;17(1):e0262444. doi: 10.1371/journal.pone.0262444 (PMC8752018; doi:10.1371/journal.pone.0262444)
Supplement: S1 Table — (DOCX) [file pone.0262444.s001.docx]

**S1 Table. 27 core cities in the YRDUA.**

|  | **Anhui Province** | **Jiangsu Province** | **Zhejiang Province** | **Shanghai City** |
| --- | --- | --- | --- | --- |
| City | Anqing | Changzhou | Hangzhou | Shanghai |
|  | Chizhou | Nanjing | Huzhou |  |
|  | Chuzhou | Nantong | Jiaxing |  |
|  | Hefei | Suzhou | Jinhua |  |
|  | Maanshan | Taizhou-JS | Ningbo |  |
|  | Tongling | Wuxi | Shaoxing |  |
|  | Wuhu | Yancheng | Taizhou-ZJ |  |
|  | Xuancheng | Yangzhou | Wenzhou |  |
|  |  | Zhenjiang | Zhoushan |  |
