# Supplementary material for: Analysis of the spatio-temporal network of air pollution in the Yangtze River Delta urban agglomeration, China
Source: PLoS One. 2022 Jan 11;17(1):e0262444. doi: 10.1371/journal.pone.0262444 (PMC8752018; doi:10.1371/journal.pone.0262444)
Supplement: S2 Table — (DOCX) [file pone.0262444.s002.docx]

**S2 Table. Individual characteristics of the STN-AP in the YRDUA^a^.**

| **City** | ***N_1_*** | **Rank** | ***N_2_*** | **Rank** | ***N_3_*** | **Rank** |
| --- | --- | --- | --- | --- | --- | --- |
| Anqing | 42.308 | 19 | 0.138 | 23 | 63.415 | 19 |
| Changzhou | 73.077 | 6 | 3.139 | 6 | 78.788 | 6 |
| Chizhou | 34.615 | 26 | 0.031 | 26 | 60.465 | 26 |
| Chuzhou | 38.462 | 22 | 0.073 | 25 | 61.905 | 22 |
| Hangzhou | 80.769 | 5 | 5.829 | 3 | 83.871 | 5 |
| Hefei | 61.538 | 7 | 1.263 | 7 | 72.222 | 7 |
| Huzhou | 46.154 | 13 | 0.534 | 14 | 65.000 | 13 |
| Jiaxing | 50.000 | 10 | 0.657 | 11 | 66.667 | 10 |
| Jinhua | 46.154 | 14 | 0.418 | 15 | 65.000 | 14 |
| Maanshan | 50.000 | 11 | 0.562 | 13 | 66.667 | 11 |
| Nanjing | 80.769 | 4 | 4.682 | 5 | 83.871 | 4 |
| Nantong | 46.154 | 15 | 0.792 | 10 | 65.000 | 15 |
| Ningbo | 46.154 | 16 | 0.418 | 16 | 65.000 | 16 |
| Shanghai | 80.769 | 3 | 5.321 | 4 | 83.871 | 3 |
| Shaoxing | 42.308 | 20 | 0.194 | 21 | 63.415 | 20 |
| Suzhou | 100.000 | 1 | 11.602 | 1 | 100.000 | 1 |
| Taizhou-JS | 42.308 | 21 | 0.321 | 18 | 63.415 | 21 |
| Taizhou-ZJ | 38.462 | 23 | 0.077 | 24 | 61.905 | 23 |
| Tongling | 46.154 | 17 | 0.207 | 20 | 65.000 | 17 |
| Wenzhou | 38.462 | 24 | 0.168 | 22 | 61.905 | 24 |
| Wuhu | 57.692 | 8 | 0.898 | 9 | 70.270 | 8 |
| Wuxi | 96.154 | 2 | 9.968 | 2 | 96.296 | 2 |
| Xuancheng | 57.692 | 9 | 1.046 | 8 | 70.270 | 9 |
| Yancheng | 38.462 | 25 | 0.220 | 19 | 61.905 | 25 |
| Yangzhou | 46.154 | 18 | 0.352 | 17 | 65.000 | 18 |
| Zhenjiang | 50.000 | 12 | 0.629 | 12 | 66.667 | 12 |
| Zhoushan | 30.769 | 27 | 0.000 | 27 | 59.091 | 27 |
| Average | 54.131 | 19 | 1.835 | 23 | 69.884 | 19 |

^a^ Due to limitation of the layout, only the annual average data is displayed.
