# Supplementary material for: Analysis of the spatio-temporal network of air pollution in the Yangtze River Delta urban agglomeration, China
Source: PLoS One. 2022 Jan 11;17(1):e0262444. doi: 10.1371/journal.pone.0262444 (PMC8752018; doi:10.1371/journal.pone.0262444)
Supplement: S3 Table — (DOCX) [file pone.0262444.s003.docx]

**S3 Table. The block classification.**

| **Block I** | **Block II** | **Block III** | **Block IV** |
| --- | --- | --- | --- |
| Anqing | Changzhou | Hangzhou | Jinhua |
| Chizhou | Nanjing | Huzhou | Ningbo |
| Chuzhou | Taizhou-JS | Jiaxing | Shaoxing |
| Hefei | Yancheng | Nantong | Taizhou-ZJ |
| Maanshan | Yangzhou | Shanghai | Wenzhou |
| Tongling | Zhenjiang | Suzhou | Zhoushan |
| Wuhu |  | Wuxi |  |
| Xuancheng |  |  |  |
