# Supplementary material for: Analysis of the spatio-temporal network of air pollution in the Yangtze River Delta urban agglomeration, China
Source: PLoS One. 2022 Jan 11;17(1):e0262444. doi: 10.1371/journal.pone.0262444 (PMC8752018; doi:10.1371/journal.pone.0262444)
Supplement: S4 Table — (DOCX) [file pone.0262444.s004.docx]

**S4 Table. The spillover effect between blocks.**

| **Block** | **Receive relationship** | | **Overflow relationship** | | **Expected internal relationship ratio** | **Actual internal relationship ratio** |
| --- | --- | --- | --- | --- | --- | --- |
|  | **Inside the block** | **Outside the block** | **Inside the block** | **Outside the block** |  |  |
| Block I | 40 | 53 | 40 | 4 | 27% | 91% |
| Block II | 26 | 25 | 26 | 44 | 19% | 37% |
| Block III | 40 | 23 | 40 | 82 | 23% | 33% |
| Block IV | 20 | 37 | 20 | 8 | 19% | 71% |
