# Supplementary material for: Analysis of the spatio-temporal network of air pollution in the Yangtze River Delta urban agglomeration, China
Source: PLoS One. 2022 Jan 11;17(1):e0262444. doi: 10.1371/journal.pone.0262444 (PMC8752018; doi:10.1371/journal.pone.0262444)
Supplement: S5 Table — (DOCX) [file pone.0262444.s005.docx]

**S5 Table. QAP correlation results of influencing factors on spatial correlation of air pollution.**

| **Variables** | **Obs Value** | **Sig.** | **Average** | **Std Dev** | **Minimun** | **Maximun** | ***P*>=0** | ***P*<=0** |
| --- | --- | --- | --- | --- | --- | --- | --- | --- |
| *G* | 0.324 | 0.000 | -0.004 | 0.111 | -0.385 | 0.312 | 0.000 | 1.000 |
| *E* | 0.496 | 0.000 | -0.002 | 0.122 | -0.369 | 0.484 | 0.000 | 1.000 |
| *U* | 0.292 | 0.009 | 0.000 | 0.127 | -0.436 | 0.420 | 0.009 | 0.992 |
| *I* | 0.301 | 0.007 | 0.001 | 0.123 | -0.420 | 0.406 | 0.007 | 0.994 |
| *C* | 0.343 | 0.000 | -0.003 | 0.100 | -0.311 | 0.367 | 0.000 | 1.000 |
| *W* | -0.181 | 0.054 | -0.002 | 0.108 | -0.365 | 0.330 | 0.954 | 0.054 |
| *T* | 0.207 | 0.016 | 0.000 | 0.110 | -0.456 | 0.260 | 0.016 | 0.988 |
| *S* | -0.148 | 0.099 | -0.002 | 0.108 | -0.428 | 0.297 | 0.911 | 0.099 |
| *D_1_* | 0.513 | 0.000 | 0.001 | 0.051 | -0.169 | 0.211 | 0.000 | 1.000 |
| *D_2_* | -0.335 | 0.000 | -0.001 | 0.045 | -0.195 | 0.149 | 1.000 | 0.000 |
| *D_3_* | -0.256 | 0.000 | -0.001 | 0.061 | -0.194 | 0.219 | 1.000 | 0.000 |
