# Supplementary material for: Analysis of the spatio-temporal network of air pollution in the Yangtze River Delta urban agglomeration, China
Source: PLoS One. 2022 Jan 11;17(1):e0262444. doi: 10.1371/journal.pone.0262444 (PMC8752018; doi:10.1371/journal.pone.0262444)
Supplement: S6 Table — (DOCX) [file pone.0262444.s006.docx]

**S6 Table. QAP regression results of influencing factors on spatial correlation of air pollution.**

| **Variables** | **Un-Stdized Cofficient** | **Stdized Cofficient** | **Sig.** | **Prob. A** | **Prob. B** |
| --- | --- | --- | --- | --- | --- |
| Intercept | 0.060 | 0.000 |  |  |  |
| *G* | 0.106 | 0.108 | 0.039 | 0.039 | 0.961 |
| *E* | 0.319 | 0.320 | 0.000 | 0.000 | 1.000 |
| *U* | 0.076 | 0.085 | 0.084 | 0.084 | 0.916 |
| *I* | 0.121 | 0.124 | 0.039 | 0.039 | 0.962 |
| *C* | 0.031 | 0.032 | 0.339 | 0.339 | 0.662 |
| *W* | -0.118 | -0.119 | 0.033 | 0.967 | 0.033 |
| *T* | 0.079 | 0.081 | 0.082 | 0.082 | 0.918 |
| *S* | -0.027 | -0.022 | 0.349 | 0.651 | 0.349 |
| *D_1_* | 0.358 | 0.358 | 0.001 | 0.001 | 0.999 |
| *D_2_* | -0.106 | -0.108 | 0.394 | 0.606 | 0.394 |
| *D_3_* | -0.192 | -0.130 | 0.723 | 0.277 | 0.723 |
